# Editorial: Web tools for modeling and analysis of biomolecular interactions Volume II

**DOI:** 10.3389/fmolb.2023.1190855

**Published:** 2023-06-09

**Authors:** Jessica Andreani, Brian Jiménez-García, Masahito Ohue

**Affiliations:** ^1^ Université Paris-Saclay, CEA, CNRS, Institute for Integrative Biology of the Cell (I2BC), Gif-sur-Yvette, France; ^2^ Zymvol Biomodeling, Barcelona, Spain; ^3^ Department of Computer Science, School of Computing, Tokyo Institute of Technology, Kanagawa, Japan

**Keywords:** web tools, biomolecular interactions, macromolecular structure, web server, protein-DNA, molecular visualization, protein mutational study, coarse-grain

Here we present the second volume of *Web Tools for Modeling and Analysis of Biomolecular Interactions*, a Research Topic focused on showcasing excellent recent (or recently updated) web-based tools and databases in the field of computational modeling and analysis of biomolecular interactions.

Web-based tools have been of paramount importance in the development of many different scientific fields in the past decades. They represent an easy-to-use and accessible portal to a rich ecosystem of methods, and they offer several advantages over their stand-alone counterpart versions. For example, web-based tools do not require special skills for installation, only a modern web browser is needed to access them or they can even be interconnected via different well-established protocols. Next, we present the different tools that were published in this Research Topic.

PROT-ON (Koşaca et al.) provides a user-friendly web interface to systematically scan mutations at a protein-protein interface based on an input 3D structure of the complex. It returns a set of mutations found to be most significantly enriching or depleting. It also displays a set of plots representing the computationally probed mutational landscape of the interface. In this way, PROT-ON is likely to be helpful to biologists aiming to modulate a given protein-protein interaction.

The ProDFace (Pal et al.) web tool offers to dissect protein-DNA interfaces from an input 3D structure. It provides detailed information about interface contacts, in particular hydrogen bonds, interface geometry and evolutionary conservation of interface residues. This information is especially useful when evaluating modeled protein-DNA structures or developing predictive tools.

The pyDockDNA (Rodríguez-Lumbreras et al.) web server is a user-friendly web-based tool for modeling protein-DNA complexes starting from their unbound 3D structures. It applies a rigid-body docking protocol specially designed for protein-DNA complex prediction, and it is also capable of using residue-restraints to enrich the predicted models. The top 10 predicted models are displayed in 3D on the browser, and results of the protocol including the top 100 predicted models can be downloaded for further evaluation and study.

The UNRES server (Ślusarz et al.) is improved and extended for physics-based coarse-grained simulations of polypeptide chains. Updates include an optimized code, a recent scale-consistent variant of the UNRES force field, and an expanded application scope to data-assisted simulations with restraints from NMR and XL-MS measurements. The server can run small jobs directly or prepare input data for larger jobs using standalone UNRES installations. While coarse-grained simulations provide longer time scales, accuracy is lower. However, data-assisted simulations help reduce the impact of force-field inaccuracy.

The Stmol (Nápoles-Duarte et al.) component provides a convenient way to render interactive molecular visualizations in web applications developed in the Streamlit framework. With only a short block of Python code, developers can include visualizations of protein and ligand structures in their bioinformatics, cheminformatics or materials science tools. From the end-user point of view, Stmol does not require prior expertise. Stmol has already been successfully integrated into several web applications.

In summary, this second volume of the *Web Tools for Modeling and Analysis of Biomolecular Interactions* Research Topic is the living proof of a dynamic community of researchers who actively contribute with superior well-designed and actively developed tools to Open Science. As the editors of this Research Topic, we would like to thank all researchers contributing to this Research Topic for their engagement towards Open Science best values.

